# The Evolutionary Psychology of War: Offense and Defense in the Adapted Mind

**DOI:** 10.1177/1474704917742720

**Published:** 2017-12-14

**Authors:** Anthony C. Lopez

**Affiliations:** 1 School of Politics, Philosophy and Public Affairs, Washington State University, Vancouver, WA, USA

**Keywords:** warfare, coalitional psychology, politics, international relations, political psychology, collective action, risk-taking, sex differences

## Abstract

The study of warfare from an evolutionary perspective has expanded rapidly over the last couple of decades. However, it has tended to focus on the ancestral origins, prevalence, and instruments of war rather than adaptationist analyses of its underlying psychology. I argue that our evolved coalitional psychology may contain a set of distinct evolved heuristics designed specifically for offensive and defensive coalitional aggression. Data from two survey experiments are presented, in which subjects were given scenarios depicting offensive or defensive aggression and were told to make decisions, for example, regarding their willingness to participate in the conflict, their opinions of others who did not choose to participate, and their expectations benefit. The results indicate that humans do indeed distinguish readily between these two domains and that their willingness to participate, as well as their emotional responses toward others, is highly contingent upon this informational cue in adaptively relevant ways. In addition, and consistent with parental investment theory, data reveal a range of sex differences in attitudes toward coalitional aggression in the two conflict domains. Beyond the study of warfare, this project has implications for our understanding of the relationship between individual behavior and group dynamics, as well as for our understanding of the mechanisms by which the psychological framing of political events can lead to important social outcomes.

Debate on the evolution of war has typically been hamstrung by ambiguities regarding its ancestral origins and prevalence. Although the nature of ancestral environments is not unknowable, it is often incomplete, requiring integration of various lines of evidence across disciplines such as primatology, archaeology, and anthropology. This has produced two opposing arguments regarding the evolutionary origins of warfare. One group of scholars argues that we cannot infer the existence of warfare prior to direct archaeological evidence of its effects and therefore that warfare probably emerged no earlier than about 50,000 years ago (Fry, 2007). In contrast, others argue that dating the emergence of warfare to the earliest direct evidence of weapons or fortifications is to falsely conflate the instruments of warfare with the occurrence of warfare itself (LeBlanc & Register, 2003). In combination with research on coalitional violence in nonhuman primates, these scholars acknowledge a longer evolutionary history of warfare (Allen & Jones, 2014; Gat, 2006; Wrangham & Peterson, 1996).

In parallel to this disagreement, scholars also dispute the very nature of war—that is, how should war be defined? While some scholars prefer a more restrictive definition that requires a degree of social organization and weaponry, others embrace a broader view of warfare, such as “events in which coalitions of members of a group seek to inflict bodily harm on one or more members of another group” (Bowles, 2009, p. 1294). However, consensus on the essential nature of war is fundamentally unimportant. More important are our answers to the following questions: (1) What forms of coalitional violence were evolutionarily recurrent? (2) What selection pressures did these forms establish? and (3) Has our evolved coalitional psychology been designed to reason adaptively about these forms? Upon finding answers to these questions, we can then investigate the ways in which these evolved mechanisms shape the way humans reason about, create, and respond to modern and evolutionarily novel environments. From this perspective, whether we refer to evolutionarily recurrent forms of coalitional violence as “warfare” is ultimately beside the point.

These adaptationist questions are necessary because, absent methodological innovations, there are only so many ways to interpret existing evidence of ancestral environments. Future research may reveal more ancient markers of human warfare; however, direct evidence of warfare does not fossilize well. Therefore, even if warfare is as evolutionarily old as some argue, it is unlikely to be reflected in the fossil record, and archaeology alone will necessarily be insufficient for concluding the matter on its own. Nevertheless, if we are to take adaptationism seriously, then scholarship has so far largely ignored one crucial source of evidence about ancestral environments: the information processing structure of our own evolved psychology. Just as the functional design of the human eye reveals information about the visual landscapes in which human perception evolved, so the design of our coalitional psychology reflects the statistical regularities of the coalitional landscape in which we evolved—including the prevalence and form of coalitional violence or warfare. Our adapted mind is a window into the ancestral past (Cosmides & Tooby, 1987; Pinker, 1997; Williams, 1966).

Nonevolutionary theories of war are important, myriad, and useful. While it is beyond the scope of this study to review these here, it is sufficient to note that these theories tend to emphasize the formative pressures of resource competition, social stratification, social organization, weaponry, and threat environment in helping to explain both the origins and patterns of human warfare over time (Keeley, 1996; Kelly, 2000; Levy & Thompson, 2010, 2011; Otterbein, 2004; Wright, 1983). New models give greater weight to cultural evolutionary pressures, in which norms and practices within groups spread across groups as a consequence of the intensity of intergroup conflict over time.

There are many evolutionary theories of dyadic aggression (Archer, 2006; Parker, 1974; Sell, Tooby, & Cosmides, 2009), and there have been some influential attempts to explore human coalitional aggression, or warfare, as an evolutionary product (Alexander, 1979; Gat, 2006; LeBlanc & Register, 2003; Lopez, 2016; Potts & Hayden, 2008; Rosen, 2005; Thayer, 2004; Wrangham & Glowacki, 2012). More recently, there is renewed effort in explaining some dynamics of social behavior as products of group selection and multilevel selection, which often take as given, rather than seek to explain, an ancestral history of coalitional violence. In addition to these analyses, behavioral ecologists have sought to outline the conditions and dynamics of coalitional competition (Boone, 2009). Yet few apply an explicitly adaptationist approach to human coalitional aggression, or warfare, in order to explore and map the psychological decision-making mechanisms shaped by these pressures.^1^ One exception is the framework established by Tooby and Cosmides (1988) and elaborated by McDonald, Navarrete, and Van Vugt (2012), which is known as the “risk contract of war,” which outlines some of the collective action challenges of ancestral warfare that must be overcome in order for the initiation of coalitional violence to have been adaptive and reflected in our evolved psychology. The framework established below builds on these efforts in order to derive hypotheses regarding the features of the coalitional environment to which our minds should attend during coalitional violence, as well as how our evolved psychology shapes preferences contingent upon the presence of those features.

Adaptationist hypotheses on the psychology of human warfare do not necessarily dispute the relevance of variables emphasized by nonevolutionary theories of warfare, nor do they diminish the importance of cultural evolution in shaping the coalitional landscape over time. Rather, evidence of a specialized psychology of warfare helps us to understand how the mind tracks and processes these cues in the environment.

## Preliminary Theoretical Considerations on Adaptationism and War

The central claim of the following studies is that distinct forms of coalitional aggression constituted selection pressures that have resulted in psychological adaptations designed to reason adaptively within these domains. These two forms of coalitional aggression are offensive and defensive aggression—or more generally, taking versus holding territory or resources (Durham, 1976; Jervis, 1978). Before establishing the theoretical framework and deriving hypotheses, however, it is necessary to establish two preliminary theoretical considerations that are central to almost any adaptationist investigation of warfare. First, recognition that the human practice of coalitional aggression, or “war,” is neither singular nor static; and second, despite the many forms of coalitional aggression in which humans have engaged over time, there may yet be some underlying computational dynamics that are general to most or all of these forms. I discuss each briefly before turning to the present study.

### War is neither singular nor static

Human coalitional aggression is as old as it is diverse in form. One common distinction is between raids and battles (Keeley, 1996; Mathew & Boyd, 2011; Wrangham & Glowacki, 2012). Raids tend to occur by stealth, incorporate an element of surprise, and involve numerical asymmetries in which attackers largely outnumber victims. In contrast, battles tend to occur between groups of relatively similar size and are consequently riskier and lengthier in duration. Raiding is the more common form of coalitional violence among humans, and it is likely that it was also the more common form among ancestral groups. Raiding dynamics resemble the chimpanzee model of warfare, though imperfectly, since chimpanzee raids seem to involve less risk to participants than human raids (Wrangham & Glowacki, 2012). Because the computational demands, as well as the constituent risks and benefits of these two forms of coalitional violence are distinct, we might expect that they would be governed by distinct behavior regulatory systems in the mind. In other words, rather than one general “theory of war,” it could be more appropriate to develop parallel but integrated “theories of violence” that map the unique computational challenges contained within distinct domains of coalitional violence such as raiding versus battles.

### Common underlying dynamics

Although the evolutionary endurance of multiple forms of coalitional violence suggests the need for distinct adaptationst explanations of each, it is also possible that there are computational challenges that are common to all or most forms of coalitional violence (Pietraszewski, 2016). For example, if warfare is fundamentally a collective action problem, then certain computational challenges must be resolved regardless of the form that coalitional violence takes such as problems relating to the distribution of risk and the value-eroding effects of free riding. The collective action component of warfare has been noted historically and in popular narratives (Hedges, 2003), and it is also evident psychologically, particularly in both lab and field studies that repeatedly demonstrate the within-group motivational consequences of between-group conflict (Gneezy & Fessler, 2011; Puurtinen & Mappes, 2009). In short, the simple cue of between-group conflict activates a host of motivational and inferential output from adaptive psychological mechanisms designed to organize and focus members within groups in conflict. There is no evidence that this effect is not general across all forms of conflict, but its full scope has not yet been established.

Analysis thus proceeds by first examining the collective action dynamics that are likely common across multiple forms of warfare. This set of computational problems then forms the framework within which separate mechanisms for offensive and defensive coalitional behavior can be derived. In short, an understanding of the dynamics that are common *across* forms of warfare allows a clearer perspective on how *unique* forms of war may alter or calibrate these dynamics.

## War Is a Collective Action Problem

War is fundamentally a cooperative endeavor (Harcourt & Waal, 1992; Price, Cosmides, & Tooby, 2002; Ridley, 1997), which suggests that many of the elements necessary for the successful prosecution of coalitional aggression (both offensive and defensive) will be similar to those required for collective action in general. Collective action problems typically entail at least two broad components, each of which represents a class of reproductive challenges. The first encompasses an individual’s *own* decision to participate, and the second relates to an individual’s ability and willingness to enforce the participation of *others*. The individual decision to participate in violence is both somatically and reproductively risky, though not unconditionally so. It is by now widely recognized that the regulation of one’s own participation in coalitional aggression should therefore be sensitive to adaptively relevant cues that reveal information relating to, for example, relative formability, the distribution of risk, the value of the collective goal, and the probability of success (Parker, 1974; Sell et al., 2009; Tooby & Cosmides, 1988). For example, the link between individual formability and out-group aggression has been experimentally demonstrated and even helps to predict support for aggressive foreign policy in modern contexts (Sell et al., 2009).

The second component of collective action consists of a class of adaptive challenges relating to within-coalition enforcement. In war, as well as many instances of nonaggressive collective action, punishment is a necessary ingredient for sustained cooperation (Boyd & Richerson, 1992; Fehr & Gachter, 2000). Recruitment and maintenance of coalition size and cohesion is also of critical importance. Below the threshold of mechanized militarized conflict, in combination with the element of surprise and an accommodation for skill, numerical superiority would have almost certainly guaranteed victory in ancestral environments (Wrangham, 1999). Therefore, successful coalitional aggression requires adaptations not only for eliminating free riding but also for labor recruitment.^2^

In general, the elimination of free riders and the recruitment of labor can be accomplished through mechanisms designed either to punish or reward the behavior of others, but the activation of these sentiments may follow ancestrally adaptive logics. For example, Michael Price, Cosmides, and Tooby (2002) have demonstrated that “reward sentiments” were unlikely to have been designed by natural selection for the purpose of eliminating free riders, since participants (who are already incurring the cost of contributing) would have to offer free riders a reward for changing their behavior (causing them to contribute) that is higher than the net benefit they already get by not contributing. This puts contributors at an even greater fitness disadvantage than they experience as a result of the free riding of others. Instead, Price et al. argue that “punitive sentiments” operate more narrowly and efficiently for the purpose of eliminating free riders in collective action by directly reversing the fitness differential that exists between free riders and participants, and thus, punitive sentiments should be most intensely felt by participants, since the fitness differential is greatest between free riders and participants. This is consistent with biological interpretations of tit-for-tat punishment, or negative reciprocity, in nonhuman animals (Clutton-Brock & Parker, 1995). In short, as an anti-free rider strategy, punitive sentiment succeeds where reward sentiments are exploitable. Evidence from public goods games in economics has produced similar results, in which reward in the absence of punishment almost always leads to the unraveling of collective action (Hilbe & Sigmund, 2010; Sefton, Shupp, & Walker, 2006).

Although reward sentiments are inefficient at solving the free rider problem, Price et al. (2002) provide evidence that reward sentiments may function to recruit labor, and they show that the motivation to reward participants is positively associated with one’s expected gain from collective action, independent of one’s willingness to participate. Regardless of whether you participate in collective action (e.g., perhaps you are sick or otherwise unable), if you expect to benefit, and the benefit offered to recruits does not exceed your expected benefit from the collective action, selection should favor a motivation to reward recruits so long as this recruitment is likely to lead to coalitional success.

This brief analysis offers an initial sketch of the class of adaptive problems that essentially characterize the balance of resolve between initiators and recruits that is managed, in part, through the manipulation of punitive and reward sentiments.^3^ Now that some of the basic features of the collective action framework have been outlined, I consider offense and defense as two domains of coalitional violence that have distinct effects upon collective action in war, and I derive hypotheses to be tested.

### Offense and defense in the adapted mind

The distinction between offensive and defensive aggression is an old consideration in the study of warfare. While Sun Tzu considered offense the more effective form of warfare, Clausewitz argued that defense was the easier strategic position. Of course, Sun Tzu and Clausewitz wrote in the background of vastly different military and strategic environments. Modern international relations scholars approach the question by considering the effects of offensive and defensive military technology on incentives to go to war (Biddle, 2001; Brown, Coté, Lynn-Jones, & Miller, 2004; Jervis, 1978). The distinction between offensive and defensive forms of aggression has been considered by students of animal behavior (Durham, 1976; Heckhausen & Heckhausen, 2010) and has recently been given greater attention by behavioral scientists of many stripes (Böhm, Rusch, & Gürerk, 2016; De Dreu et al., 2016; Lopez, 2012; Rusch, 2013, 2014a, 2014b, 2014c). Given the prominence of these two forms of violence in human history and given the recognition of similar distinctions in nonhuman animals, it is worth considering whether these two forms of violence would have represented distinct adaptive problems. Thus, if psychological adaptations exist that are designed to contingently respond to the unique challenges posed by defensive and offensive coalitional aggression, then we must begin by considering the evolutionarily recurrent and reproductively significant differences that may have existed between these two domains.

Ancestrally, one difference between offensive and defensive coalitional behavior would have been that the benefits of successful offensive aggression include nonpublic goods to a relatively greater degree than those derived from successful defensive aggression (Durham, 1976; Lopez, 2012; Rusch, 2014b; Tooby & Cosmides, 1988).^4^ For example, a successful defense that repels an intruder and denies it access to territory and resources confers the benefits of this success (e.g., security, preservation of resources, threat removal, status) to the entire group. However, offensive aggression that is successful in taking resources from another group is more likely to include a nonnegligible amount of privatized resources, including perishable food and mating opportunities, which asymmetrically accrue to the participants rather than the group as a whole.^5^ In short, the major distinction between offense and defense from which hypotheses are derived is that successful defensive coalitional aggression mirrors the structure of a public good, and successful offensive coalitional aggression confers benefits that are relatively more privatized among those who choose to participate in violence. Thus, these two types of aggression can be distinguished in terms of their respective ancestral cost–benefit structure.

In defense, an out-group initiates aggression, and the challenge is to respond quickly and effectively. The preexisting bonds characterized by kin-based hunter-gatherer bands facilitate a quick defensive response to external aggression. Additionally, intergroup dynamics such as *shadenfreude* and reduced empathy for outgroupers in competitive environments fuel the motivational component of defensive action. Thus, there is a preexisting bias toward group support that motivates participation in defensive coalitional aggression. In contrast, the initiation of offensive aggression shifts the burden of coalitional action onto those who would initiate aggression. Unlike the case of defense, in which benefits are distributed relatively symmetrically and publicly, in offensive aggression benefits accrue asymmetrically and often privately to participants, and the relevant consideration in this domain will be that of *personal* gain, rather than *group* gain. Thus, the first two hypotheses propose that the calibration of behavior-regulatory mechanisms in the brain for participation in coalitional aggression will depend upon the domain of aggression. In offense, willingness to participate should be predicted by perceived personal gain, while in defense, willingness to participate should be predicted by group gain (Hypotheses 1 and 2, respectively).

Differences in the ancestral cost–benefit structure of offense and defense suggest that punitive and reward sentiments toward nonparticipants should operate differently in these two conflict domains. Price et al. (2002) conducted a preliminary test of this conjecture but found that punitive sentiment toward nonparticipants was predicted by one’s willingness to participate in both defensive and offensive contexts; that is, they found no effect of conflict domain. However, if the gains from offense are less of a public good, then nonparticipants in this domain are not free riders and should not be targeted by punitive sentiments, in contrast to the authors’ findings. One possible explanation for this puzzle is that the offense–defense manipulation was not effective. Therefore, I restructure the manipulation to more clearly reflect the offense–defense distinction (discussion on design below) and test the following hypothesis: Punitive sentiment by participants toward nonparticipants should be triggered by willingness to participate in defense but not in offense (Hypothesis 6).^6^

I have argued that the domain of offensive aggression shifts the motivational burden of coalitional action to the initiators and that their enforcement of the participation of others is a function of their ability and willingness to absorb the costs of this enforcement on others. Also as noted earlier, Price et al. (2002) provide evidence that reward sentiment toward participants is triggered specifically by one’s expected gain from coalitional aggression, independent of one’s actual level of participation. This will remain true in defense, due to its public good nature; however, since participants more easily privatize benefits in offensive aggression, reward sentiment toward participants in offense will be triggered by one’s willingness to participate (Hypothesis 7). In other words, in offense, nonparticipants who reward participants but who (necessarily) receive no benefit would be at a fitness disadvantage. Only participants would have an interest in rewarding other participants, to the extent it solves the labor recruitment problem. Thus, we have reason to expect that the domain of coalitional aggression (offense vs. defense) will contingently and adaptively regulate: (1) the willingness of individuals to participate and (2) their ability and motivation to enforce the participation of others through the positive and negative inducements of reward and punishment, respectively.

### Sexual dimorphism in warfare

Sex differences may be adaptively contingent upon the domain of conflict. Sexual selection and parental investment theory have proven remarkably successful at predicting and explaining the zoological diversity of sexual dimorphism and have proven useful for explaining a range of sex differences in humans (Archer, 2006; Buss & Schmitt, 1993; Daly & Wilson, 1988; Ermer, Cosmides, & Tooby, 2008; Hudson & Boer, 2002; Johnson et al., 2006; Mesquida & Wiener, 1999; Wilson & Daly, 2002). This theoretical framework, in combination with supporting evidence from primate studies (Manson & Wrangham, 1991; Wrangham & Peterson, 1996), leads us to expect that, all else equal, males should be more willing to participate in coalitional aggression than females.

On the one hand, sexual selection and parental investment theory explain why males, given lower levels of parental investment, have been equipped by natural selection with the phenotypic weapons of aggressive intrasexual (male vs. male) competition (Symons, 1979; Trivers, 1972). On the other hand, it is a direct consequence of this investment asymmetry that *coalitional* aggression has been reproductively advantageous for males more than for females (McDonald, Navarrete, & Van Vugt, 2012; Tooby & Cosmides, 1988). At the outset, therefore, we should expect that, regardless of whether the domain is offensive or defensive, males should be more willing to participate in coalitional aggression than females (Hypothesis 3).

In terms of expected benefit, however, given that successful defense confers more in the way of public goods than successful offense, all group members experience the main benefits of successful defensive aggression regardless of sex. Despite the fact that investment asymmetries render female coalitional aggression a high-cost, low-benefit endeavor, this does not mean that aggression avoidance should be psychologically instantiated as an unconditional strategy in females. Despite the fact that the fitness benefits to females of engaging in coalitional violence are often low relative to males, the threat of invasion by a male coalition is a particularly costly prospect for females (McDonald et al., 2012; Van der Dennen, 1995; Van Vugt, 2009), such that we are therefore unlikely to observe sex differences in expected benefit in defense (Hypothesis 4). In other words, males and females will equally appreciate the value of prevailing in defensive aggression. In contrast, we are likely to observe greater relative levels of expected benefit by males than by females in offense (Hypothesis 5). This is a direct reflection of the fact that females have more to lose and less to gain, either directly or indirectly, from *offensive* coalitional aggression since the benefits of such action remain privatized among participants, even accepting the fact that the material benefits of such coalitional action may be shared among close family or allies.

### Hypotheses

**Hypothesis 1:** In *offense*, willingness to participate will be triggered by expectations of personal benefit, even controlling for expectations of group benefit.
**Hypothesis 2:** In *defense*, willingness to participate will be triggered by expectations of group benefit, even controlling for expectations of personal benefit.
**Hypothesis 3:** Males will be more willing than females to participate in *both* offensive and defensive coalitional aggression.
**Hypothesis 4:** There will be no sex difference in expected benefit from *defensive* coalitional aggression.
**Hypothesis 5:** Males will expect greater benefit than females from *offensive* coalitional aggression.
**Hypothesis 6:** In *defense, but not in offense*, punitive sentiment toward nonparticipants will be triggered by one’s willingness to participate.
**Hypothesis 7:** In *defense*, reward sentiment toward participants will be triggered by one’s expected benefit from coalitional aggression, but in *offense*, reward sentiment toward participants will be triggered by one’s willingness to participate.

## Study 1

### Design

Hypotheses were tested with an experiment-based survey in 2007. The subject population consisted of 195 undergraduate students (83 female) at the University of California, Santa Barbara, mostly political science majors between the ages of 18 and 39. The survey presented both an offensive and defensive scenario. Each subject read both scenarios, and the order in which the scenarios were presented was counterbalanced across subjects to minimize order effects. Thus, the conflict domain manipulation (offense/defense) followed a within-subjects implementation.

This experiment-based survey is adapted from and expands upon an earlier survey conducted by Price et al. (2002); however, in this study, the scenarios are revised and new survey questions are added. The survey design in Price et al., while not focused on examining separate psychologies of offense and defense per se, nevertheless tested for this and found no supportive evidence. This may be partly due to the fact that their offensive scenario depicted retaliatory action against a foreign country, which could easily have triggered defensive rather than offensive psychology. For example, in Price et al., subjects read the following offensive and defensive vignettes:

Offense: Imagine that a few years from now, several oil-rich Middle Eastern countries get together and decide that to increase profits, they will dramatically raise the price of their oil. This price increase devastates US industry and causes high inflation in the USA. US gas prices triple, and several US oil companies go bankrupt. After talks with these Middle Eastern countries fail, the USA declares war on them. But war was unexpected, so the USA has allowed its army to get relatively small, and it must start drafting US citizens in order to have a chance of victory. How would you feel about this war?

Defense: Imagine that a few years from now, the Russian people elect a new, warlike dictator who claims that Alaska should rightfully belong to Russia. Under this dictator, Russia invades and conquers Alaska. There is good evidence that Russia also intends to conquer more US territory, in addition to Alaska. In response to this invasion, the USA declares war on Russia. But because this war was unexpected, the USA has allowed its army to get relatively small, and it must start drafting US citizens in order to have a chance of winning this war. How would you feel about this war?

Two major changes were made to these vignettes. First, both scenarios are rewritten in a nomadic context 1,000 years ago. This is done in order to “decouple,” as best as possible, modern views toward war in the context of nation-states and our beliefs regarding those states from the operation of the psychological adaptations themselves. For example, the above vignettes invoked the prospect of war with Russia and with Middle Eastern countries, all of which represent culturally significant out-groups with unique histories of interaction that are likely to affect the calibration of psychological adaptations in confounding ways (e.g., inspiring especially high motivation to engage in violence against a Russian invasion, as well as increased susceptibility to outrage in the offensive scenario given the intersection of oil and politics in the Middle East).

Second, the offense scenario specifically was reorganized in order clarify that the question is whether to initiate unprovoked aggression. In the offense scenario utilized by Price et al. (2002), it is possible that subjects interpreted the scenario as one of retaliation for price hikes. In that instance, assuming real differences between offense and defense psychologies, it is likely that a defense psychology would have been activated, instead of an offense psychology. In sum, vignettes were utilized that engaged subjects in hypothetical scenarios, and the offense scenario specifically was reworked in order to ensure that the decision was whether to initiate unprovoked aggression against an out-group. It is worth noting that the use of hypothetical scenarios for exactly this purpose in experimental designs is increasingly common (Mitchell, Tetlock, Newman, & Lerner, 2003). The new scenarios are:

“Defense: Imagine that you and your friends and kinsmen are the members of a nomadic horse-riding people, the Pathans, in the year 1050. You and your group are tough and strong, but your life is meager. You cook over dung fires, drink fermented mare’s milk, sleep in yurts under skins, and freeze every winter. Your ancestral territory is on the dry steppe, but you also border on the prosperous Chinese province of Sinkiang. You have heard that the governor of the province is ambitious and intends to extend his control into your territory. If you lose your grasslands, you and your family will have nothing. The governor has assembled a war party, which has launched a series of nighttime raids on your people. Some of your women have been kidnapped, and some of your men were killed. You hear rumors that another raid is going to happen tonight. You and your friends and fellow tribesmen begin to discuss whether you should form a war party to repel the raiders.”
“Offense: Imagine that you and your friends and kinsmen are the members of a nomadic horse-riding people, the Pathans, in the year 1050. You and your group are tough and strong, but your life is meager. You cook over dung fires, drink fermented mare’s milk, sleep in yurts under skins, and freeze every winter. Your ancestral territory is on the dry steppe, but you also border on the prosperous Chinese province of Sinkiang. You sometimes enter these cities to trade, but the Chinese men and women laugh at your poor clothes, your dirt, and your lack of refinement. You do notice that the men are short, weak, and cowardly, despite the fact that they give themselves airs. The women are also beautiful and dressed in the best silks.
You and your friends have heard that the Mongols to the North had attacked and taken over several cities and are now wealthy and powerful. The Chinese troops had run like rabbits. You and your friends and fellow tribesmen begin to discuss whether you should form a war party to seize one of the neighboring cities.”

Immediately following each scenario, subjects were asked a series of questions relating to their willingness to participate, the extent to which they personally expected to benefit, anticipated benefit to the group, expectations that others would join, desire to punish free riders, and desire to reward participants. Answers to each question were self-reported on a 7-point Likert-type scale from *strongly disagree* (1) to *strongly agree* (7). An identical battery of questions followed each scenario with minor modifications where contextually appropriate.

## Results

### Hypotheses 1 and 2

Willingness to participate is predicted by expected benefit to the group (“If my group succeeded…it would benefit us as a group”) in defense (Hypothesis 1) but by expected personal benefit (If my group succeeded…it would benefit me personally”) in offense (Hypothesis 2). Hypotheses 1 and 2 are expected if defense is a public good, while the benefits of offense tend to be subject to privatization.

**Figure 1.**
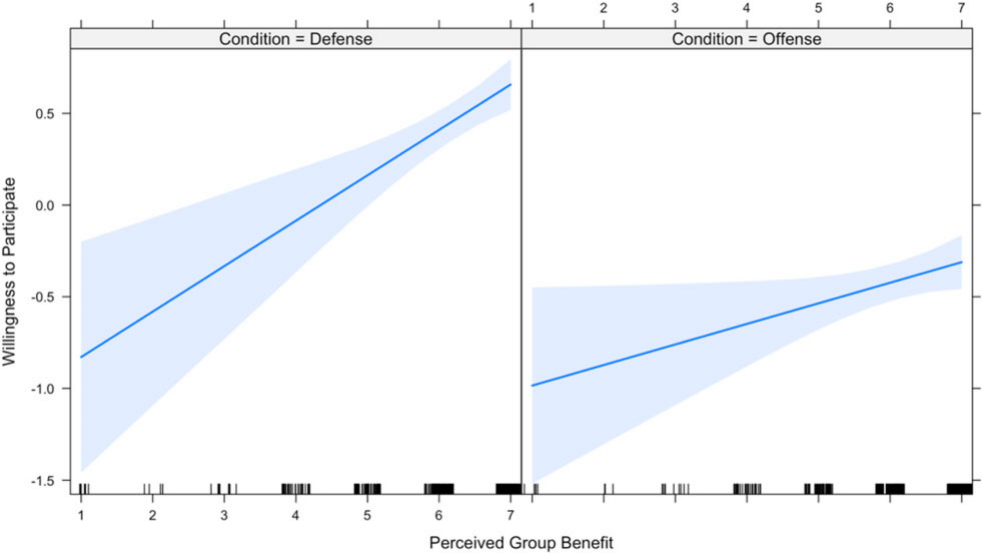
Relationship between perceived group benefit and participation, by conflict type.

**Figure 2.**
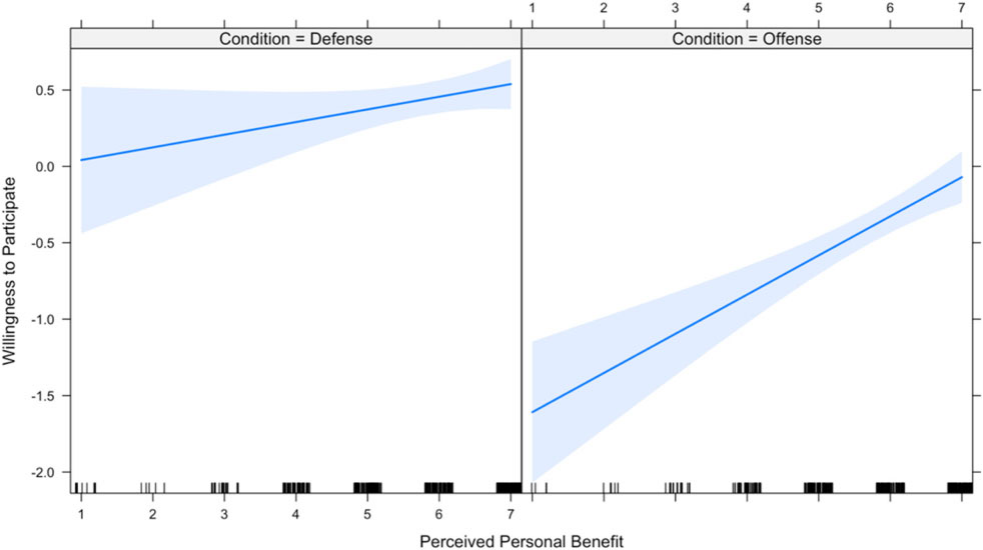
Relationship between perceived personal benefit and participation, by conflict type.

The first two hypotheses predict that the effect of expected group benefit and expected personal benefit on one’s willingness to participate is conditional upon the domain of offense versus defense (hereafter, “condition”). Therefore, these two hypotheses were tested using a linear mixed effects regression model that includes each of the main independent variables as well as interaction terms with condition on each of the independent variables. Due to high inter-item reliability across the four measures of participation, the dependent variable is a new variable that scales the four measures. Results (Table 1) show that both interaction terms are in the predicted direction; however, the moderating effect of condition on expected group benefit narrowly misses significance at the conventional .05 level. Figures 1 and 2 plot the interactions and visually display the moderating effect of condition on expected group benefit and expected personal benefit, respectively. All analyses (in both Studies 1 and 2) were computed using R programming software 3.4.1.

**Table 1.** Hypotheses 1 and 2.

	Dependent Variable
Independent Variable	Participation
Condition	−0.999***
	(.329)
Expected group benefit	0.248***
	(.061)
Expected personal benefit	0.083
	(.051)
Condition × Group Benefit	−0.136*
	(.077)
Condition × Personal Benefit	0.173**
	(.067)
Constant	−1.546***
	(.293)
Observations	390
Log likelihood	−408.788
Akaike information criteria	833.575
Bayesian information criteria	865.180

*p < .1. **p < .05. ***p < .01.

### Hypothesis 3

Males are more willing than females to participate in both offensive and defensive aggression.

A mixed effects regression model showed that condition did not moderate the effect of sex on willingness to participate (Table 2). In other words, males are indeed more willing to participate than females, and, as predicted, that relationship was not significantly altered by condition.

**Table 2.** Sex Differences in Participation.

	Dependent Variable
Independent Variable	Participation
Sex (0 = M; 1 = F)	−0.561**
	(.223)
Condition (0 = Def; 1 = Off)	−1.750***
	(.167)
Sex × Condition	−0.238
	(.255)
Constant	6.187***
	(.146)
Observations	390
Log likelihood	−711.594
Akaike information criteria	1,435.188
Bayesian information criteria	1,458.923

*p < .1. **p < .05. ***p < .01.

### Hypotheses 4 and 5

Males expect greater benefit than females from offensive, but not from defensive, coalitional aggression.

I examined two measures of perceived benefit: “If my group succeeded in repelling the invaders/seizing a city, it would *benefit me personally*” and “If my group succeeded in repelling the invaders/seizing a city, it would *benefit us as a group*.” Again, mixed effects regression was used to examine the moderating effect of condition upon the relationship between sex and both types of expected benefit.

**Figure 3.**
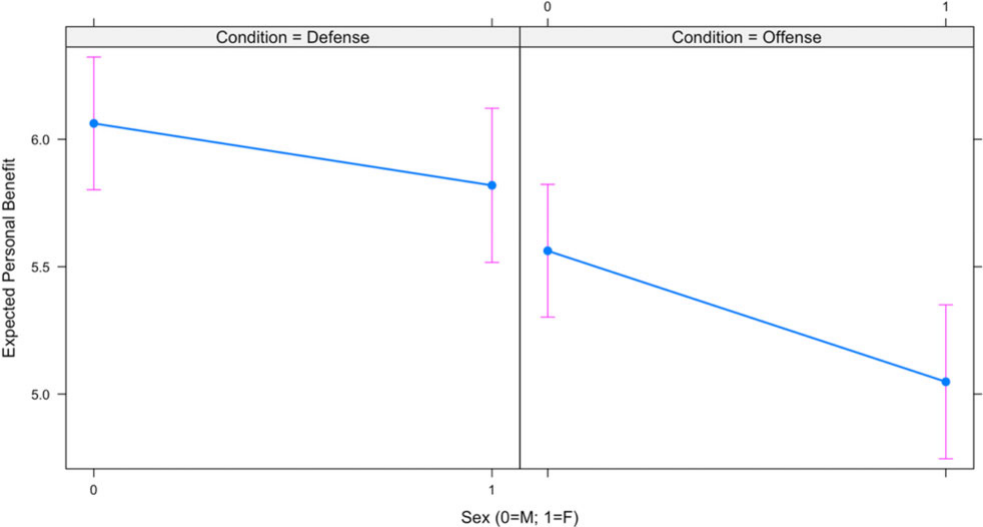
Sex differences in perceived personal benefit.

**Figure 4.**
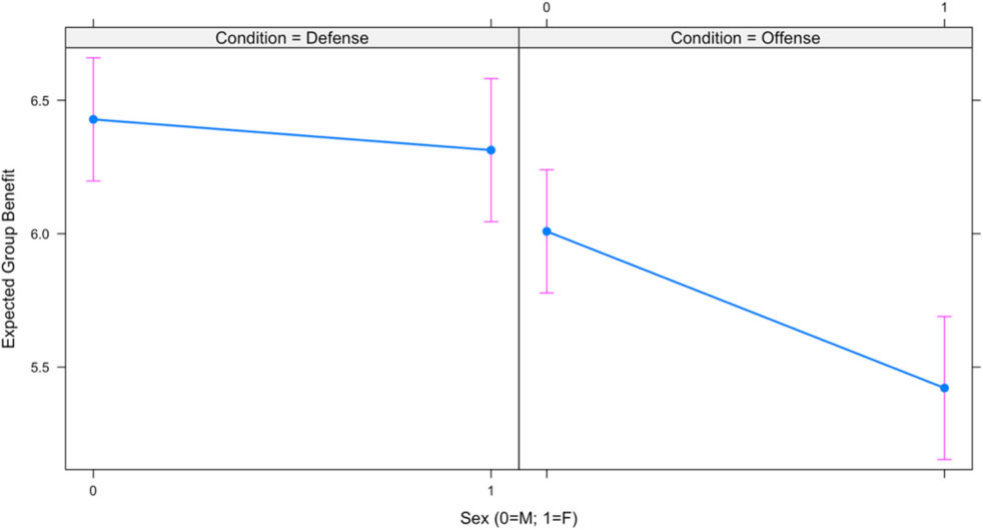
Sex differences in perceived group benefit.

Results for two linear mixed effects regression models are presented in Table 3. The first model examines sex differences in perceived personal benefit by condition, and the second model examines sex differences in perceived group benefit by condition. As shown, the moderating effect of condition on sex was in the predicted direction but not statistically significant in the first model (*b* = −0.271, *p* = .22); however, the moderating effect of condition on sex was in the predicted direction and statistically significant (*b* = −0.472, *p* = .02) in the second model. The interactions for each model are depicted in Figures 3 and 4, respectively. Thus, although statistical significance is not uniformly met, the relationships are in the predicted direction.

**Table 3.** Sex Differences in Perceived Benefit

	Dependent Variable	
Independent Variable	Expected Personal Benefit	Expected Group Benefit
	(1)	(2)
Condition	−0.500***	−0.420***
	(.144)	(.132)
Sex	−0.243	−0.115
	(.203)	(.180)
Sex × Condition	−0.271	−0.472**
	(.221)	(.202)
Constant	6.063***	6.429***
	(.132)	(.117)
Observations	390	390
Log likelihood	−669.552	−626.577
Akaike information criteria	1,351.103	1,265.155
Bayesian information criteria	1,374.838	1,288.890

*p < .1. **p < .05. ***p < .01.

### Hypothesis 6

Willingness to participate will track punitive sentiment (“If one of my fellow tribesmen did not participate, I’d think they should be punished”) in defense but not in offense.

**Figure 5.**
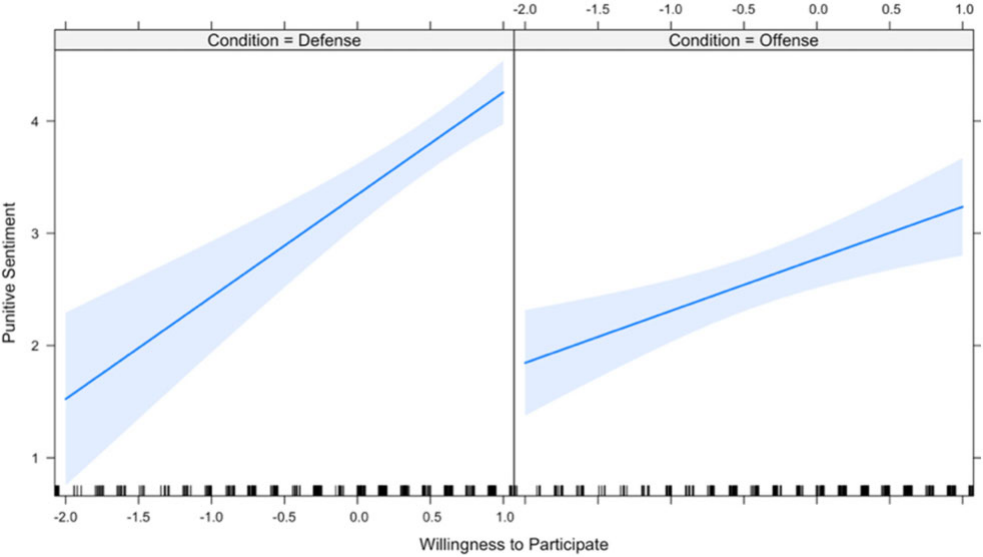
Relationship between participation and punitive sentiment, by conflict type.

**Figure 6.**
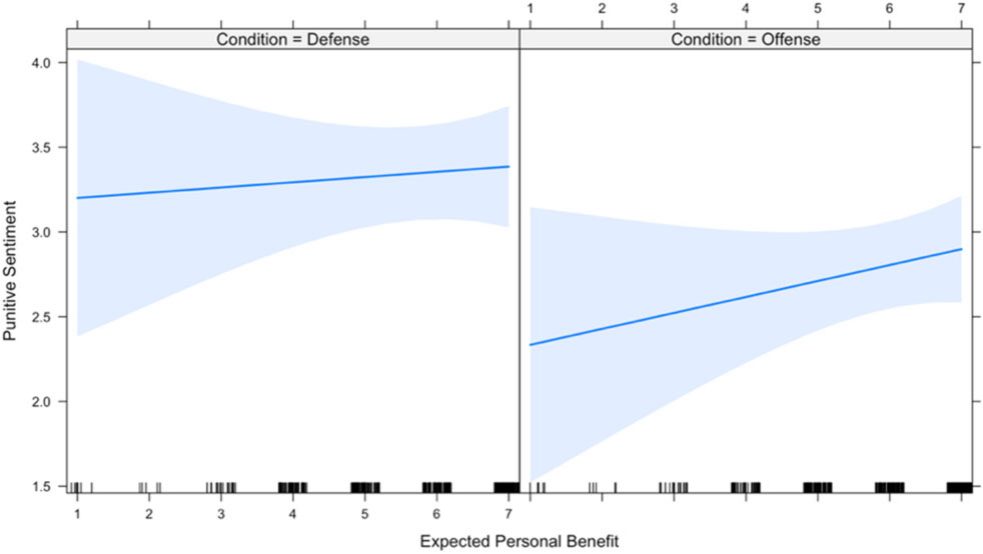
Relationship between perceived personal benefit and punitive sentiment, by conflict type.

Price et al. (2002) demonstrated that punitive sentiment was predicted by willingness to participate, even when controlling for expected benefit, and that there was no statistically significant relationship between expected benefit and punitive sentiment when controlling for willingness to participate. However, I hypothesized that since offense is less of a public good than defense, condition should moderate the effect of willingness to participate on punitive sentiment, since nonparticipants are not free riders, strictly speaking. In short, I hypothesized a replication of Price et al. in defense but not in offense. A mixed effects regression model included two interactions to test whether condition moderates the effect of participation or expected benefit on punitive sentiment. Results (Table 4, Model 1) indicate that condition indeed moderates the effect of participation on punitive sentiment (*b* = −0.447, *p* = .01). In other words, Price et al.’s study is replicated in defense, but the effect is weakened in offense. In contrast, and consistent with Price et al. (2002), expected benefit had no significant effect on punitive sentiment either on its own (*b* = 0.031, *p* = .71) or when interacted with condition (*b* = 0.063, *p* = .55). Figures 5 and 6 illustrate each interaction.

**Table 4.** Predicting Punitive (Hypothesis 6) and Reward Sentiment (Hypothesis 7).

	Dependent Variable	
Independent Variable	Punitive Sentiment	Reward Sentiment
	(1)	(2)
Condition	−0.930	1.064
	(.628)	(.669)
Willingness to participate	0.910***	0.364**
	(.151)	(.156)
Expected personal benefit	0.031	0.338***
	(.085)	(.089)
Condition × Participation	−0.447**	0.600***
	(.179)	(.191)
Condition × Personal Benefit	0.063	−0.123
	(.107)	(.114)
Constant	3.170***	2.740***
	(.496)	(.514)
Observations	390	390
Log likelihood	−711.983	−714.101
Akaike information criteria	1,439.965	1,444.202
Bayesian information criteria	1,471.570	1,475.807

*p < .1. **p < .05. ***p < .01.

### Hypothesis 7

Perceived benefit to self should predict sentiments for rewarding participants in defense, but willingness to participate should predict reward sentiment (“If one of my fellow tribesmen did participate, I’d think they should be rewarded”) in offense. Again, a replication of Price et al. (2002) is expected in defense when gains are public, but in offense, since victory is not a public good, nonparticipants who reward participants but who receive no benefit from victory would be at a fitness disadvantage. Only participants would benefit from rewarding other participants. Therefore, condition should moderate the effect of participation and perceived benefit on reward sentiment; specifically, participation should trigger reward sentiment in offense but not in defense, while perceived benefit should trigger reward sentiment in defense but not in offense.

**Figure 7.**
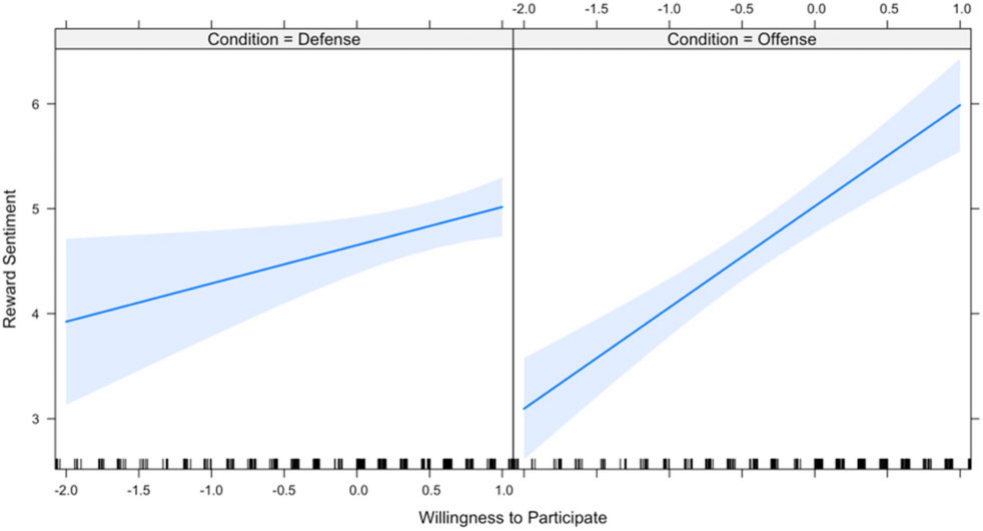
Predicting reward sentiment.

**Figure 8.**
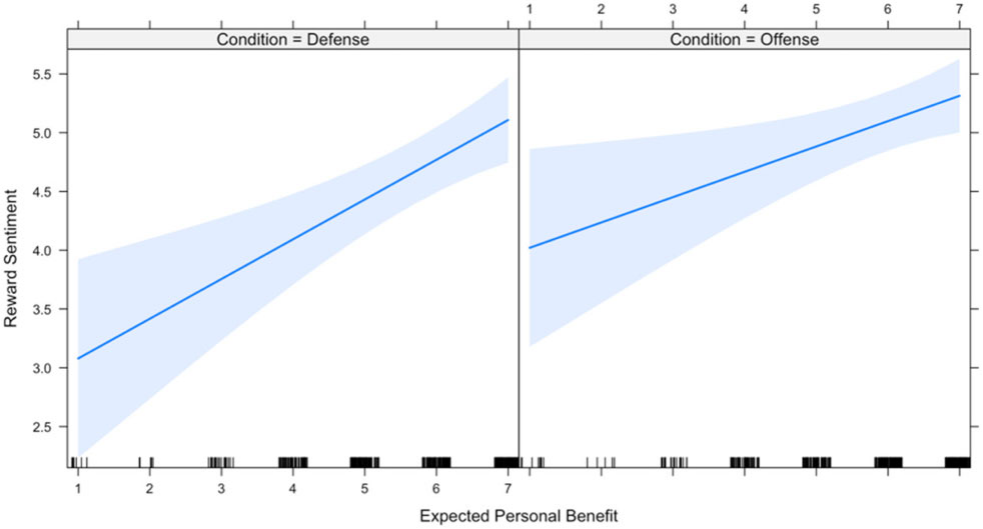
Predicting reward sentiment.

Results of a mixed effects regression (Table 4, Model 2) show strong support for the moderating effect of condition on participation (*b* = 0.600, *p* = .00). However, although the moderating effect of condition on perceived benefit was indeed in the predicted direction, it failed to reach statistical significance (*b* = −0.123, *p* = .28). Resulting interactions are depicted in Figures 7 and 8.

It is noteworthy that in offense, *both* benefit to self and willingness to participate predict reward sentiment. One possible explanation for these findings derives from the earlier discussion of the distinction between defense and offense as public versus private goods. To the extent that the benefits from successful offense are mostly private goods but include a nonnegligible amount of public goods (e.g., status, deterrence), it follows that benefit to self might still predict reward sentiment in offense as in defense. For example, if there are at least some public benefits that accrue as a consequence of offensive coalitional aggression, then it follows that there may some individuals who (1) expect the benefits of action will make them better off, either directly or through benefits that inhere to the group, and (2) for some reason may wish to participate but are not able to (e.g., sickness, injury). In these instances, the (real or imagined) existence of a public good severs the narrow link between participation and perceived personal benefit, such that even nonparticipants can expect benefits—and when they do, they should act to reward participants to the extent that the value of their donation does not outweigh their on-average gain.

## Discussion of Study 1

Hypotheses 1–5 are generally supported by the data and help to reveal the effect of conflict domain (offense/defense) on the operation of mechanisms designed in part to regulate within-group participation in intergroup conflict. Results supported the broad pattern of evidence in favor of male aggressiveness, but again, conflict domain adds granularity to this relationship. For example, males are more willing to participate in both offense and defense, and males are less pessimistic than females about the group benefits of offensive aggression. Importantly, there was no meaningful sex difference in the perceived personal or group benefits of defensive aggression. This supports new research that downplays a purported unconditional preference for aggression in males and reveals one aspect of the domain specificity of such preferences.

Hypotheses 6 was supported—offensive aggression weakens the relationship between participation and punitive sentiment due to the fact that nonparticipants in offense are not free riders as they are in defense. Nevertheless, one must remember that it is most likely that successful offensive coalitional aggression confers many types of benefits, some of which may indeed take the form of a public good. In other words, both defensive and offensive coalitional aggression sometimes confer some types of public goods when successful (e.g., elevated status, deterrence), but offensive coalitional aggression typically comes with the *added* premium of privatized benefits to the participants. Thus, future research should further investigate the cue structure of these two domains of aggression. Although there is already fruitful movement in this direction, the inferential possibilities are myriad.

Hypothesis 7 received mixed support—willingness to participate strongly predicted reward sentiment in offense, as predicted, but perceived personal benefit predicted reward sentiment in both offense and defense. In other words, in defense, only perceived benefit significantly predicts reward sentiment toward nonparticipants, but in offense, both perceived benefit and willingness to participate predict reward sentiment.

## Study 2

### Punitive Sentiment as Labor Recruitment Device

One of the implications of the previous study is that punitive sentiment may be designed, at least in part, to recruit labor. This is somewhat intuitive since punitive sentiment, by reversing fitness differentials between participants and free riders in the present, may serve to motivate participation in the future by deterring would-be defectors. Relatedly, the challenge of recruiting labor should be central to our coalitional psychology, especially given the ancestral imperative of relative numbers for intergroup aggression. Therefore, in Study 2, hypotheses are derived and tested regarding the effect of perceived labor levels on punitive sentiment toward nonparticipants and willingness to participate in offensive and defensive aggression.

Coalitional aggression is predominantly a male activity (McDonald et al., 2012; Mesquida & Wiener, 1999; Van der Dennen, 1995; Van Vugt, 2009; Wrangham & Peterson, 1996); however, collective defense is also a public good, in which the fruits of victory are neutral with respect to sex (Rusch, 2013). Therefore, while the previous sex difference of greater male participation is perceived when labor is sufficient, it is possible that female participation and recruitment will increase when labor is insufficient due to the public nature of defensive victory, placing female participation at or near male participation. In contrast, since offensive coalitional aggression is largely characterized by goods privatized among the participants—who are overwhelmingly male—it is unlikely that female participation and recruitment will be affected by perceived labor levels. Males, in contrast, will seek to optimize participation and recruitment to the needs of the offensive endeavor, rather than maximize participation and recruitment outright, as in defense. If participation and recruitment are cast as conditional strategies, then we can hypothesize simply that males are conditional strategists in offense but unconditional strategists in defense, while females are conditional strategists in defense but unconditional in offense (Table 5). With respect to labor levels, this yields the following predictions regarding individual participation:

**Table 5.** Predicted Levels of Punitive Sentiment and Participation.

Conflict Type	Labor Sufficient	Labor Insufficient
Defense	Males high/females low	Males high/females high
Offense	Males low/females low	Males high/females low

Hypotheses on willingness to participate:

**Hypothesis 8:** In defense, male participation should remain high and unaltered by perceived labor conditions; female participation should be greater when labor is perceived to be insufficient.
**Hypothesis 9:** In offense, male participation should be highly incentivized by “nearly there” labor levels; female participation should remain low and unaltered by perceived labor levels.

Furthermore, given the link between participation and punitive sentiment, the intuitive expectation is that recruitment—in the form of punitive sentiment toward nonparticipants—should follow the same pattern. In other words, we have the following parallel predictions regarding punitive sentiment:

Hypotheses on punitive sentiment:

**Hypothesis 10:** In defense, male punitive sentiment should remain high and unaltered by perceived labor conditions; female punitive sentiment should be greater when labor is perceived to be insufficient.
**Hypothesis 11:** In offense, male punitive sentiment should be highly incentivized by “nearly there” labor levels; female punitive sentiment should remain low and unaltered by perceived labor levels.

Before proceeding to tests and results, it is worthwhile to further elaborate the nature of the sex-specific conditional strategies in war. The divergence in strategies becomes apparent when we consider the marginal effects of hypothetical changes to labor levels in defense and offense. Each is examined in turn.

Defense, being a public good, is such that every additional unit of labor added to the effort only further decreases the cost–benefit ratio faced by each participating individual, since more labor only reduces costs in an environment in which benefits are public and fixed.^7^ Furthermore, given a greater baseline willingness of males to participate in coalitional aggression than females, there is no obvious theoretical reason why female participation should be as high or higher than male participation in defense when available labor is already sufficient. When labor is insufficient, however, it is possible that we may see a greater increase in female participation, particularly if this is viewed as necessary for victory and likely to bring about that outcome.

In offense, every additional unit of labor to the effort does not invariably reduce the cost–benefit ratio experienced by participants. For example, when labor is insufficient for the successful execution of offensive coalitional aggression, participants (initiators) have a unique and powerful interest in recruiting labor since they have identified that the initiation of offensive aggression would benefit them. In contrast, however, when labor is sufficient^8^ for the execution of offensive coalitional aggression, each additional unit of labor actually increases the cost–benefit ratio, assuming that the benefits are private and fixed.^9^ For example, if there are 50 units of resources to be plundered from out-group X and 10 individuals are required in order to surprise and overwhelm out-group X and steal away with those resources, then each individual potentially receives 5 units as reward (plunder) on average.^10^ However, if 15 more individuals are added to the effort, then the cost–benefit ratio is effected in the following way. On the cost side, the fact that the raiders have more than doubled in size reduces the risk to which each is exposed (although it was already sufficiently low), while also increasing the probability of success (although it was already sufficiently high). Furthermore, as numbers expand, the offensive coalition at some point must inevitably lose the element of surprise by virtue of size alone, which was among the most critical factors determining success in ancestral landscapes. On the benefit side, with 25 total individuals participating in the raid, each individual now only receives two resource units on average. Thus, the expectation is that males will optimize participation and recruitment in offense according to the demands of the situation. Again, given the predominantly male character of coalitional aggression, the expectation would be that the above analysis explains male but not female motivation in offensive war. Instead, this perspective would predict female participation and recruitment to remain low in offense regardless of any perceived change in labor levels.

A further note is warranted regarding male participation and recruitment in offense. The above analysis suggests that there is likely a quadratic relationship in offense between participation and perceived labor levels. In other words, at *extremely* low labor levels, it may make little sense to invest the time and effort necessary to build a coalition; instead, what is more likely is that the effort will fizzle upon lack of interest and motivation.^11^ However, as labor levels begin to increase (i.e., as more become interested and willing), it becomes increasingly worthwhile to engage in the endeavor until a critical threshold is reached, after which point the coalition members experience diminishing marginal returns per capita as more participants join, essentially “turning off” participation and recruitment.^12^ As Rusch (2013) notes,

…once the critical threshold required to overpower the enemy combatants is reached, the individual share of loot acquired through raiding out-groups is likely to decrease with the number of warriors participating in the raid, while the individual chances of survival for defenders are likely to increase with every additional man participating in the defense. (p. 975)

In addition to the rapid diffusion of benefits that extra labor causes in offense, there is the additional problem of coalition management, which only becomes increasingly challenging with ever larger coalitions. In contrast, defense poses more of a basic coordination problem due to its immediate and public nature, and therefore, the extra labor is less of a burden and more of an asset.

## Design

One hundred and eighty-eight undergraduate subjects participated in this study, of which 74 were male and 114 were female. The within-subjects design of offensive and defensive vignettes and questions was maintained. A new between-subjects manipulation was incorporated that altered the design in the following way. Although all subjects read an offense and a defense vignette as before, in this round, each subject reads vignettes in which either success was unlikely if more people do not join (labor insufficient) or success was likely even if more people do not join (labor sufficient). This was accomplished by adding a couple of sentences to the end of each vignette describing one or the other labor condition. For example, half of the subjects received a survey in which both vignettes (offense *and* defense) described coalitional aggression that was unlikely to succeed if more people did not join, while the other half received a survey in which both vignettes (again, both offense and defense) described coalitional aggression that would probably succeed even if more people did not join. The manipulations added to the end of the vignettes are:

### Defense

Labor Insufficient: Although a large war party can be successful against the invaders, if many of the reluctant people do not join the war party, its current size may be insufficient and therefore unlikely to repel the invaders. This may result in your tribe’s collective defeat at the hands of the foreigners.

Labor Sufficient: Although many are reluctant to join the war party, the current amount of committed volunteers should be enough to repel the invaders.

### Offense

Labor Insufficient: Although a large war party can be successful in seizing a city, if many of the reluctant people do not join the war effort, your war party, at its current size, will be unlikely to seize a city. Instead, it will probably be repelled by the foreigners and be forced to return empty-handed.

Labor Sufficient: Although some are reluctant, the current amount of eager volunteers should be enough to seize a city, even if the reluctant people do not join the war party.

## Results

### Hypotheses on Punitive Sentiment

Punitive sentiment is predicted to be a function of the three-way interaction between conflict type, labor condition, and sex. As a prelude to investigating this claim, and for exploratory purposes, a mixed effects regression model that includes all predictor variables without interactions is presented (Table 6). Results indicate that willingness to participate, labor condition, and conflict type all have a strong and statistically significant effect on punitive sentiment. Substantively, punitiveness toward nonparticipants is positively linked with participation, enhanced when labor is particularly needed, and greater in defense than in offense. The model also shows that sex as well as expectations of personal and group benefit produced weak and nonsignificant effects upon punitive sentiment.^13^

**Table 6.** Predicting Punitive Sentiment.

	Dependent Variable
Independent Variable	Punitive Sentiment
Labor condition	.299***
	(.112)
Participation	.181***
	(.029)
Conflict type	−.264***
	(.077)
Expected personal benefit	.027
	(.046)
Expected group benefit	−.073
	(.057)
Sex	.134
	(.115)
Constant	−.627**
	(.290)
Observations	376
Log likelihood	−476.354
Akaike information criteria	970.707
Bayesian information criteria	1,005.904

*p < .1. **p < .05. ***p < .01.

**Figure 9.**
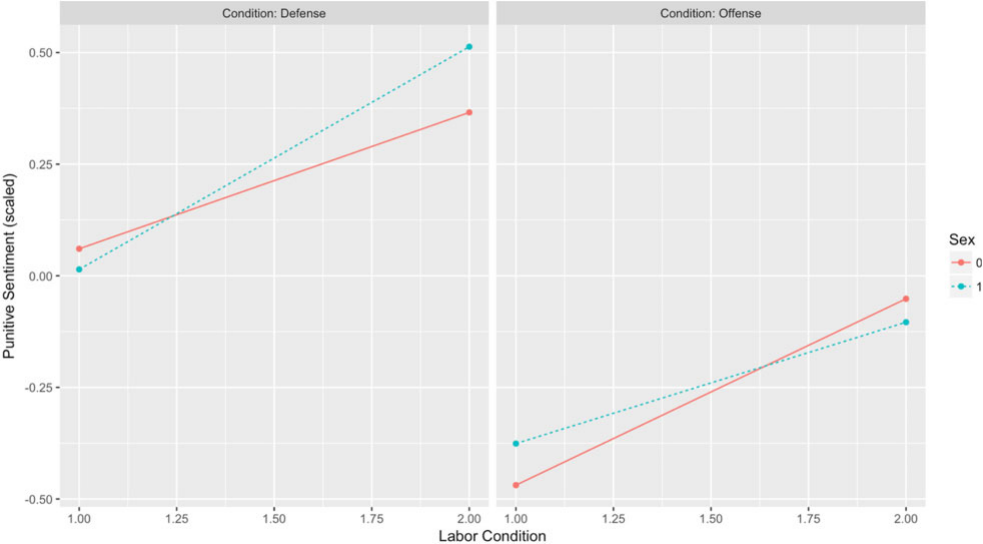
Punitive sentiment: Conflict Type × Labor Condition × Sex.

Is punitive sentiment a function of a three-way interaction between conflict type, labor condition, and sex? Results indicate that although the interaction term was in the predicted direction, the effect failed to reach statistical significance (*b* = −0.339, *p* = .22). Table 7 presents the results of the interaction model, and Figure 9 illustrates the observed three-way interaction.

**Table 7.** Predicting Punitive Sentiment (Interactions).

	Dependent Variable
Independent Variable	Punitive Sentiment
Labor condition	.305
	(.223)
Sex	−.046
	(.203)
Conflict type	−.529^***^
	(.154)
Labor Condition × Sex	.193
	(.286)
Labor Condition × Conflict Type	.112
	(.218)
Sex × Conflict Type	.139
	(.199)
Labor Condition × Sex × Conflict Type	−.339
	(.280)
Constant	.060
	(.158)
Observations	376
Log likelihood	−492.702
Akaike information criteria	1,005.405
Bayesian information criteria	1,044.486

*p < .1. **p < .05. ***p < .01.

### Hypotheses on Willingness to Participate

**Figure 10.**
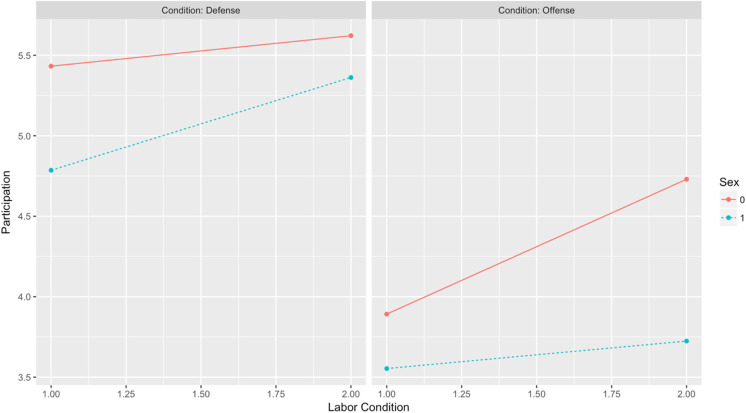
Willingness to participate: Conflict Type × Labor Condition × Sex.

Individual participation is predicted to be a function of the three-way interaction between conflict type, labor condition, and sex. Results of a mixed effects regression (Table 8) support the predicted interaction, although narrowly missing statistical significance at the conventional .05 level (*b* = −1.05, *p* = .07). Figure 10 illustrates the observed interaction.

**Table 8.** Predicting Willingness to Participate.

	Dependent Variable
Independent Variable	Participation
Labor condition	0.189
	(.404)
Conflict type	−1.541***
	(.313)
Sex	−0.647*
	(.368)
Labor Condition × Conflict Type	0.649
	(.443)
Labor Condition × Sex	0.387
	(.519)
Conflict Type × Sex	0.308
	(.404)
Labor Condition × Conflict Type × Sex	−1.054*
	(.569)
Constant	5.432***
	(.286)
Observations	376
Log likelihood	−724.853
Akaike information criteria	1,469.706
Bayesian information criteria	1,508.787

*p < .1. **p < .05. ***p < .01.

### Post Hoc Analysis

**Figure 11.**
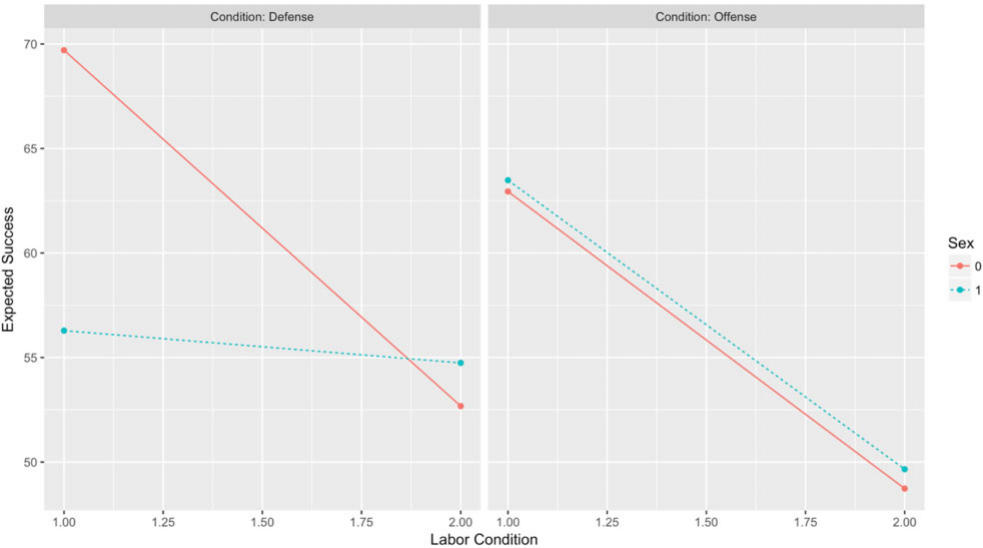
Expectations of success: Conflict Type × Labor Condition × Sex.

**Figure 12.**
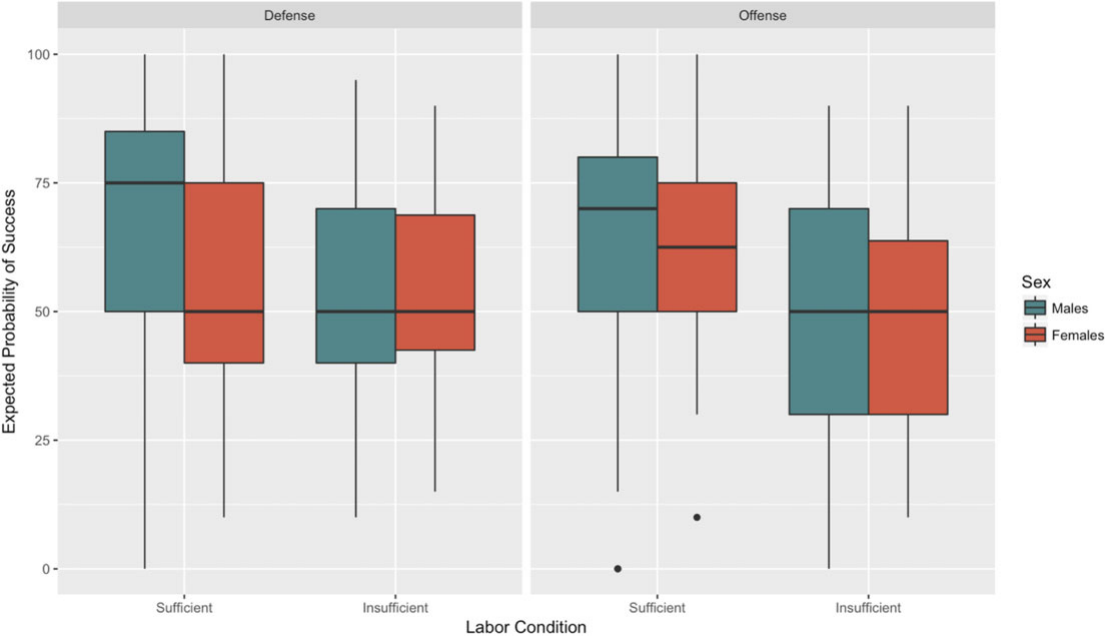
Sex differences in expected success.

No hypotheses were established regarding the effect of perceived labor levels on the expected probability of success. However, given the widely accepted importance of relative numbers for coalitional aggression that is often cited in the literature (e.g., Wrangham, 1999), it would seem straightforward to expect, all things equal, that expectations of success should be greater in conditions in which labor is perceived as sufficient rather than insufficient. In other words, the effect of labor condition on expected success should be strong and significant. However, a mixed effects regression model returned a strong and significant *interaction* between conflict type, labor condition, and sex, indicating that although more labor was tied to greater expectations of success for males in both offense and defense, the same was not also true for females (*b* = −15.09, *p* = −.05). This surprising result will be addressed in the discussion below. Figures 11 and 12 illustrate the observed interaction.

## Discussion of Study 2

### Punitive Sentiment

As predicted by Price et al. (2002), punitive sentiment is closely tied to one’s participation level; however, as argued above, it is also tightly linked with conflict type and labor condition, further indicating that it does indeed play a role in labor recruitment as predicted. Contrary to predictions, however, there was little evidence that sex predicted punitive sentiment either on its own or in interaction with other variables.^14^ Importantly, as found in Study 1 and in previous studies, sex does predict willingness to participate reasonably well. Taken together, what this suggests is that the punitive sentiment system is sensitive to cues of participation rather than to the sex of the participant, since it is one’s participation, rather than sex, per se, that is more immediately indicative of the cost–benefit profile of the coalitional context. Nevertheless, due to the relatively small sample size and the fact that the three-way interaction between conflict type, labor condition, and sex was nevertheless in the predicted direction despite missing statistical significance, the idea is worth examining further. However, the current results offer limited support at best.

### Willingness to Participate

Results support the prediction that participation is a function of the three-way interaction of conflict type, labor condition, and sex. In offense, labor conditions affected strongly predicted male participation, while in defense, labor conditions affected female participation. In contrast to punitive sentiment, results provide greater support for the predictions regarding individual participation.

Lastly, we must consider the surprising finding that female appraisals of the probability of success remained unaffected by labor levels in defense. One explanation for this finding may be rooted in the psychology of self-deception. As mentioned above, females bear a unique cost in war, and it may be that pessimism is a form of *negative* illusion designed to compel the participation of others, if not specifically male participation. Of course, such negative illusions find their analogue in positive illusions, or overconfidence. In this regard, it is noteworthy that males are more prone to overconfidence than females in conflict scenarios (Johnson & Fowler, 2011; Johnson et al., 2006), and it may be the case that defense—the warfare domain most threatening to females—uniquely triggers this form of negative deception. Relatedly, it may also be the case that the sex difference in appraisal in the context of defense may be facilitated by differences in mood. For example, Lerner and Keltner (2001) found that anger facilitates risk-seeking and overconfidence, while fear tends to facilitate risk avoidance and pessimism. In other words, defense may facilitate sex-specific emotional reactions—fear in females and anger in males—and to the extent that fear is linked with pessimism, this may generate undue pessimism from females in defense but not in offense, since the fear associated with invasion would not be imminent. These possibilities are purely speculative, unfortunately, and these data do not allow a test of these explanations, but there is cause for further examination.

Taken together, these findings lend support to the general conjecture that perceived labor levels contingently shape decision-making in coalitional aggression. *Individual participation* was observed to be sensitive to the interaction of conflict type, labor condition, and sex, as predicted. In contrast, although there was no three-way interaction between conflict type, labor condition, and sex on *punitive sentiment*, punitiveness was still strongly predicted by conflict type and labor condition, which is consistent with punitive sentiment playing a role in labor recruitment rather than merely reversing fitness differentials between participants and nonparticipants. Again, this suggests that it is the physical participation of the individual, rather than their sex, that the punitive sentiment system takes as the relevant cue for regulating punitiveness toward nonparticipants.

## General Discussion

Scope and limitations of the research design are discussed first, followed by a discussion of the implications of the empirical results for adaptationist theories of war as well as related concepts in the field of international relations. Since this research combines two provocative areas of research (adaptationism and warfare), some care is necessary when outlining these issues.

### Scope and Limitations of the Research Design

The current design faces limits to internal validity due to the fact that one of the main manipulations (offensive vs. defensive domains) was implemented using a within-subject survey experiment design, meaning that each subject received both manipulations, although the order of the manipulations was randomly varied in order to mitigate ordering effects. Furthermore, within-subjects designs yield a greater likelihood of committing type II errors, in which one fails to reject a false null hypothesis. In other words, within-subject manipulations may weaken a causal relationship that exists, leading the researcher to falsely conclude that the manipulation or treatment has no effect (e.g., that conflict domain has no effect on respondent choices). In the context of these difficulties, however, it is noteworthy that hypotheses regarding the offense/defense manipulation were largely supported and in the predicted direction. Nevertheless, future research could expand and amend the design to overcome this limitation.

Problems of external validity exist as well, largely due to the demographics of the subject population—almost exclusively an undergraduate population. Clearly, generalization is limited. Importantly, however, this student population is in fact the same age as most individuals in active combat and therefore may be ecologically valid for some reasons while problematic for others. Nevertheless, research must proceed in stages, in which initial tests establish proof of concept, and future studies aim to replicate findings and employ alternative methodologies that overcome these limitations, which are far from insurmountable. At no point is the argument made that any of the above results offer conclusive evidence of adaptations for warfare; rather, the findings are consistent with hypotheses that were derived from adaptationist analyses, drawing upon a range of theory and evidence, such as, but not limited to, known and inferred ancestral selection pressures. Both the assumptions upon which the model rests (e.g., ancestral conditions and the game theoretic structure of coalitional dynamics) as well as the hypotheses themselves are falsifiable. Improvements in internal and external validity require, at a minimum, further tests against alternative hypotheses, as well as basic replication and improvements in sample size and representativeness.

Another potential issue is regarding the differentiation between offensive and defensive domains of war. In this study, the distinction specifically relates to the *initiation* of violence, while acknowledging that as conflict unfolds, particularly in modern combat, the distinction between offense and defense can quickly become fluid and nebulous. Analysis is therefore restricted to the initiation of conflict, which is also often the practice of scholars of international relations (Jervis, 1978) and ethologists (Durham, 1976). The core distinction is that defense represents aggressive reaction to foreign attempts to take territory or resources, while offense represents the aggressive initiation of attempts to seize territory or resources, where resources are broadly defined as material or nonmaterial. Vignettes were constructed with attention to establishing the cues that were likely to have ancestrally correlated with each domain. For example, the defensive domain involves the imminent arrival of a foreign coalition of unknown size and strength, while the offensive domain involves the opportunity to attack and seize resources from a weak out-group/coalition. However, future research should investigate and explore the nature of the cue structure of offensive and defensive framing that trigger these distinct psychologies

The difficulty, in practice, of distinguishing between offensive and defensive aggression should not be underemphasized. However, it may be the case that the subjective ambiguity that we experience in the midst of within-group debate and discussion on the utility of foreign aggression is likely in part the *product* of the complex relationship between the two psychologies rather than the evidence of the absence of a real or useful distinction.

Another methodological consideration is the choice of a tribal context for the survey vignettes instead of a modern context. As mentioned above, this was done in order to decouple modern moral preconceptions regarding the use of force. However, one might object that in such a “tribal” environment in the absence of moral constraints, any individual should be happy to jump at the opportunity to engage in violence with reckless abandon, especially given the rewards for such action. Obviously, results indicate that this was not the case, and the fact that the hypothesized conditional strategies were supported suggests that the tribal context did not have the effect of implicitly and generally signaling that aggression was less reprehensible or more sanctioned in general. Nevertheless, future research could easily vary many aspects of the vignette in order to isolate desired contextual features, which could allow helpful investigation of how this psychology might operate in a modern institutional context.

### Final Remarks and Extensions

These studies apply an adaptationist lens to the question of human coalitional aggression or warfare. Although coalitional aggression can take many forms—some of which are enduring while others are fleeting—the conceptual challenge is to outline the underlying collective action challenges that must be solved in order for coalitional aggression to be successfully initiated. For example, humans must have been able to reason adaptively about whether to join the aggression one’s self as well as whether and how to manipulate the participation of others. Regarding the latter, past research suggests that punitive and reward sentiments play a role in limiting free-riding and recruiting labor. The current study builds on this research and examines the possibility that the operation of these systems is contingent upon the particular domain of conflict: offense or defense.

Study 1 revealed that one’s willingness to participate is tied to expectations of group benefit rather than personal benefit in defense, but the reverse was true in offense. Furthermore, conflict domain revealed patterned sex differences in both the willingness to participate and expected benefit of war. Study 2 failed to return a significant three-way interaction between conflict type, labor condition, and sex for predicting punitive sentiment; yet results did further confirm the role of punitive sentiment as a labor recruitment mechanism. However, individual participation was shown to be a function of the three-way interaction of the above factors, in partial support of the divergent conditional strategies of males and females in coalitional aggression. Importantly, the architecture of female coalitional psychology is still poorly understood and undertheorized. There is accumulating evidence—to which these studies contribute—that both men and women possess psychological adaptations for warfare and that they operate according to sexually dimorphic information processing pathways. Thus, the question is not: Which sex is more aggressive? Rather, the question is: What are the conditions under which each sex is likely to engage in aggression? Relatedly, when will these conditions be similar or different between the sexes? Future research can only broaden our understanding of this important and central puzzle, as long as we start with the right questions.

We interpret the modern world through the lens of a vast array of information processing systems in the brain that were designed for solving adaptive problems in ancestral environments. This being the case, a useful next step, upon identifying the existence and operation of psychological adaptations, is to form hypotheses regarding the ways in which these adaptations interpret modern evolutionarily novel cues.

For example, one important institution that may interact with our coalitional psychology in important ways is democracy. Under democratic regimes with broad political enfranchisement, war-minded democratic executives face an acute labor recruitment problem when they seek to initiate offensive violence (Tomz & Weeks, 2013).^15^ This magnifies the motivation (self-deceived or actual) to misrepresent offensive aggression as defensive, particularly where their expected personal benefits are great. This dynamic may also lead to interesting international consequences, as the spread of democracy may indeed be accompanied to a certain degree by an ostensible norm against the initiation of offensive aggression and the competitive framing of aggression of any type as defensive. Thus, it should come as no surprise, given the significant downstream motivational consequences of war framing, that one of the most hotly debated considerations in any conflict, ranging from civil to international war, is the rather parochial question: “Who started it?” These studies indicate that the answer to this simple but powerful question may often be sufficient to trigger a host of downstream inferences and motivations regarding one’s own participation in conflict as well as one’s feelings toward others who may disagree.

This dynamic is evident in almost any debate on the relative merits of war but is particularly well illustrated by the 2003 American invasion of Iraq. The Bush administration made the case for preventive war against the Saddam Hussein regime by claiming that Hussein intended to acquire and use weapons of mass destruction (WMD). According to the administration and its supporters, the threat was imminent and indivisible, and therefore, preventive (i.e., defensive) aggression was necessary. Their opponents, however, insisted that Hussein could be contained and that WMD-related claims were exaggerated or false. The counterargument was, therefore, diametrically opposed: War was not inevitable, the threat was not shared, and American intervention would be offensive rather than preventive. Furthermore, and quite tellingly, opponents of war accused the administration and its supporters of seeking to initiate war for the sake of private interests and gains, such as oil, rather than the public good.

This is exactly how an evolved coalitional psychology should operate in the prelude to war: Cues regarding public or private gains, for example, should loom large, and individuals should actively scan and evaluate the defensive claims of supporters for evidence of deception. Ancestrally, the costs of acting upon false beliefs regarding the nature of coalitional aggression could have generated serious fitness consequences. For example, joining offensive aggression that you falsely believe to be defensive could lead to great risk for little gain at best or in making the ultimate sacrifice for the private gain of another at worst; conversely, falsely categorizing group defense as offensive would risk not only your group’s defeat but also your own survival and reproductive future. Given that the costs of being wrong about defense are likely greater than the costs of being wrong about offense, error management may result in a psychology that is relatively susceptible to being convinced that offensive aggression is actually defensive.

Given that the labor recruitment problem is generally more easily overcome for defensive than for offensive aggression, one important corollary is established: where both labor recruitment challenges and the potential personal benefits to initiators are great, those who would seek to initiate offensive aggression should, all things equal, differentially act to misrepresent offensive aggression as defensive for the sake of “cheating” the problem of labor recruitment. This misrepresentation on behalf of initiators may even occur through self-deception, in which the greater the benefit perceived by those who would seek to initiate aggression, the more likely they are to truly believe that the initiation of aggression is necessary, either for the sake of preventing future aggression or defending the status of the group, for example.

Thus, as mentioned briefly above, it can be nearly impossible in practice to determine whether any given instance of warfare is “actually” defensive or offensive. And it may be that pure cases of either offense or defense are relatively uncommon, as any given episode of coalitional aggression may indeed contain mixed motives. However, this complexity is not an argument against the existence of separate psychologies. Rather, the complexity itself, as suggested above, is the product of the delicate interplay of these two psychologies. In other words, the reliable fuzziness of political claims in the run-up to war is likely the direct consequence of the recursive interplay of individuals attempting to resolve and enforce issues of participation and recruitment across society. In this sense, a distinction between offense and defense is indeed subjective, but the content of this subjectivity is provided by the recursive interplay of (1) a species-typical evolved coalitional psychology designed to operate according to privileged hypotheses specific to the domains of warfare, (2) unique situation-specific variables relating to intracoalition variables (size of one’s coalition) and intercoalition variables (proximity to other coalitions, past history of conflict), and (3) unique individual-level variables such as sex, personal formidability, number of siblings, personal prior history in aggression, status position relative to others, and so on. It is no surprise then that wars are fought for myriad reasons and develop in endlessly unique ways; yet, this is because of, rather than despite, an evolved psychology that is both highly conditional and deeply complex.

Importantly, separate psychologies of offense and defense may also play a role in peacetime alliance politics. For example, modern notions of “collective security” in international relations institutionalize the concept of the indivisibility of threat by asserting that a threat to one is a threat to all. The evolutionary calculus, of course, is that shared threat motivates individual willingness to support the alliance, and thus, modern security institutions essentially attempt to exploit this defense psychology in order to artificially convert a dyadic nonshared threat into a broader shared threat. Collective security is therefore effective when a defense psychology is triggered among the members, such that collective action is facilitated and deterrence against external threats is enhanced. The psychological logic behind the institutional effort is sound; however, institutions alone cannot “dictate” the experience of a shared threat to its members, which of course suggests that institutions that are built on preexisting shared group boundaries and identities are more successful at coordinating collective action than institutions that are built for the purpose of creating shared group boundaries. This is evident, for example, in the very different historical trajectories experienced by the North Atlantic Treaty Organization on the one hand and the Southeast Asian Treaty Organization on the other. In other words, institutions are often more successful when they are the *products* of shared identities, rather than when they are designed in order to *produce* them.

The existence of psychological adaptations for warfare does not allow the inference that humans are naturally war-prone and that warfare is inevitable (Lopez, 2016). Instead, humans seem to possess specialized psychological design that regulates the conditional expression of behavior in response to environmental contingencies. Furthermore, although we do not have complete information regarding the ancestral past, we can use the incomplete but growing portrait that we do have to formulate hypotheses regarding how evolved mechanisms ought to logically operate if a given selection pressure was indeed present. Although evidence accumulated from adaptive task analyses and lab and field experiments are not on their own sufficient to allow us to conclusively determine the nature of ancestral environments or psychological adaptations, the simultaneous evaluation and integration of these and other lines of evidence allows us to inch ever closer to a sharper image of the link between evolution, psychology, and behavior.

## Notes

1. I will use “coalitional aggression” synonymously with “warfare,” owing largely to the definitional concerns mentioned above. I view this as a methodological convenience rather than a definitive statement on what war fundamentally is or should be.

2. Of course, it is important to recognize that there are problems in both directions. While too few participants will compromise the probability of success, too many complicates the free rider problem and multiplies the cognitive demands of coordination. This issue is elaborated in Study 2.

3. Although a digression, there is a critical role for “leaders,” since, all else equal, adaptations designed for intracoalitional enforcement should differentially motivate individuals to engage in the punishment of free riders and the recruitment of labor when they (1) have more at stake, (2) are highly formidable, and (3) enjoy high status (Tooby & Cosmides, 1988).

4. A public good is any resource that is both nonexcludable (I cannot prevent others from using it) and nonrival (my use of it does not diminish the ability of others to benefit from it).

5. It may be, however, that in terms of the structure of benefits conferred, offensive coalitional aggression may represent more of a club good (excludable, but nonrival), while defensive coalitional aggression is more clearly a public good. Future research along these lines is forthcoming.

6. The enumeration of the hypotheses is presented out of order for the sake of verbal exposition. Hypotheses 3–5 on sex differences are presented. A full list of hypotheses immediately follows this discussion for reference.

7. I do not claim that this will be true of any collective action that yields public goods; rather, this may be particularly true of collective actions that involve violence, since, as noted in Chapter 1, one of the hallmarks of successful coalitional violence is a combination of low risk and a high probability of success.

8. “When labor is sufficient” is a shorthand way of describing the fact that, in principle, there must exist a threshold point beyond which the marginal benefits of each additional unit of labor begin to decrease. Whether or not the participants are cognitively aware of the exact threshold is a separate question; in fact, they are unlikely to be precisely aware of it even if their recruitment efforts reflect its subconscious or implicit estimation. An alternative argument is that males simply follow the heuristic that more is always better rather than run the risk that they have “cut off” recruitment prematurely. However, the diffusion of benefits combined with loss of the element of surprise and rapidly mounting coordination problems suggests that in offense, more so than in defense, there is likely to be an effort to optimize rather than maximize recruitment.

9. This is compounded by the fact that each additional member added to the coalition increases the cognitive complexity of the task of coalitional coordination (Tooby, Cosmides, & Price, 2006).

10. There are many caveats here. For example, individuals may share these rewards with others upon returning to the group; however, even in this case, this sharing is likely to occur nonrandomly. Specifically, sharing is likely to occur within kin and alliance networks and not within the group as a whole. In any event, the acquisition of the resource by the individual only enhances that individual’s inclusive fitness, regardless of whether it is ultimately shared with others.

11. In the study that follows, “extremely low” labor levels will not be examined.

12. Importantly, this dynamic applies with special force in the context of the initiation of raids rather than the decision to engage in pitched battles.

13. This survey included multiple measures of punitive sentiment that were not the subject of theory in this article. Specifically, 10 questions were asked about a hypothetical male nonparticipant that were designed to measure various forms of punitive sentiment, and 10 questions were asked about a hypothetical female nonparticipant. Since the lead question of each group of 10 questions most closely matches the question measuring punitive sentiment in Study 1 (“Some of the men/women in your tribe were reluctant to join, despite being able bodied. If one of these men/women decided not to join this war party, I believe he or she should be punished.”), I focus on these two questions. Furthermore, since these two measures exhibit high inter-item reliability (α = .81), I generated a scaled variable combining them into a single punitive sentiment variable.

14. In addition, two-way interactions between sex and conflict type as well as sex and labor condition returned null results. A two-way interaction between sex and willingness to participate was statistically significant, but the effect was so weak as to be substantively meaningless (*b* = 0.09, *p* = .03).

15. Although in the context of military institutions leaders do not necessarily face the challenge of recruiting soldiers, the labor recruitment problem is manifested in the challenge of recruiting support for violence. Ancestrally as well as today, political support must anchor the success of coalitional violence.
